# Active Bone Conduction Implant and Adhesive Bone Conduction Device: A Comparison of Audiological Performance and Subjective Satisfaction

**DOI:** 10.1055/s-0043-1777416

**Published:** 2024-03-11

**Authors:** Maria Fernanda Di Gregorio, Carolina Der, Sofia Bravo-Torres, Mario Emilio Zernotti

**Affiliations:** 1 Department of Otoneurology, Sanatorio Allende, Nueva Córdoba, Córdoba, Argentina; 2 Department of Otorhinolaryngology, Hospital Dr. Luis Calvo Mackenna, Providencia, Santiago, Chile; 3 Department of Audiology, Hospital Dr. Luis Calvo Mackenna, Providencia, Santiago, Chile; 4 Department of ENT, Sanatorio Allende, Nueva Córdoba, Córdoba, Argentina; 5 Speech Therapy School, Faculty of Medical Sciences, Universidad Nacional de Córdoba, Córdoba, Argentina

**Keywords:** aural atresia, bone conduction hearing aids, conductive hearing loss

## Abstract

**Introduction** Atresia of the external auditory canal affects 1 in every 10 thousand to 20 thousand live births, with a much higher prevalence in Latin America, at 5 to 21 out of every 10 thousand newborns. The treatment involves esthetic and functional aspects. Regarding the functional treatment, there are surgical and nonsurgical alternatives like spectacle frames and rigid and softband systems. Active transcutaneous bone conduction implants (BCIs) achieve good sound transmission and directly stimulate the bone.

**Objective** To assess the audiological performance and subjective satisfaction of children implanted with an active transcutaneous BCI for more than one year and to compare the outcomes with a nonsurgical adhesive bone conduction device (aBCD) in the same users.

**Methods** The present is a prospective, multicentric study. The audiological performance was evaluated at 1, 6, and 12 months postactivation, and after a 1-month trial with the nonsurgical device.

**Results** Ten patients completed all tests. The 4-frequency pure-tone average (4PTA) in the unaided condition was of 65 dB HL, which improved significantly to 20 dB HL after using the BCI for 12 months. The speech recognition in quiet in the unaided condition was of 33% on average, which improved significantly, to 99% with the BCI, and to 91% with the aBCD.

**Conclusion** The aBCD demonstrated sufficient hearing improvement and subjective satisfaction; thus, it is a good solution for hearing rehabilitation if surgery is not desired or not possible. If surgery is an option, the BCI is the superior device in terms of hearing outcomes, particularly background noise and subjective satisfaction.

## Introduction

Atresia of the external auditory canal affects 1 in every 10 thousand to 20 thousand live births. It is mostly present unilaterally (only 30% of the patients are affected bilaterally), in the right ear of male patients, 1 2 and it can be associated with microtia. 3 Reports from Latin America indicate that it is more prevalent there, affecting 5 4 to 21 out of every 10 thousands newborns. 5 A large percentage of these cases occur for unknown reasons, while some types are genetic and associated with craniofacial disorders. Most of the patients present with an air conduction pure-tone average (PTA) of 60dB to 70 dB, with a bone conduction (BC) PTA in the normal range. 6

The treatment of patients with atresia and microtia involves esthetic and functional aspects. Reconstructive esthetic surgery with autologous cartilage has had successful results. 7 In the functional approach to hearing loss, BC hearing aids (spectacle frames as well as rigid and softband systems) can be initially used. The disadvantages of these prostheses include limited functional gain, visibility, cosmetic unattractiveness, and pain due to pressure on the skin. 3

The adhesive BC device (aBCD) called ADHEAR (MED-EL, Innsbruck, Austria), released in 2017, 8 is another nonsurgical option. 9 The system consists of an adhesive adapter that attaches to the hairless skin behind the pinna in the mastoid area and is connected to an audio processor (AP). The adhesive adapter can only be used on healthy skin, is water-resistant, and can stay on the skin for several (3 to 7) days. 10 The AP receives the sound waves and turns them into vibrations. Clinical studies have shown that the audiological performance of the ADHEAR in quiet and noise is comparable to that of traditional BC hearing aids. However, these studies found the adhesive device to have several advantages, namely, superiority in wearing comfort, wearing time, and subjective satisfaction. 11 12 13

Bone conduction implants (BCIs) are another alternative to BC hearing aids. 14 The active transcutaneous BCI BONEBRIDGE (MED-EL) is one of the systems available. It consists of an implantable coil and transducer that convert the delivered signals into vibrations that are subsequently transmitted to the inner ear via the skull. Transcutaneous direct stimulation of the bone minimizes the risk of skin irritation and achieves good sound transmission. 15 As the BCI lies completely under the skin, it is not visible, and the complication rates are very low. 16 17 The BONEBRIDGE device has been approved for sale in the European market in 2012 for use in adults and, in 2014, for children over 5 years of age. 16

The present research is of particular interest for Latin American countries, which present the highest prevalence of outer ear malformations, greater than the average reported worldwide. 18 19 The high costs of implants are an access barrier for the most disadvantaged segments of the population.

Therefore, the aim of the present prospective and multicentric study was to evaluate the audiological benefits and subjective satisfaction with a BCI hearing system in patients with conductive hearing impairment over a period of one year, and to compare these results with the benefits obtained with an aBCD in the same group of users.

## Materials and Methods

**Subjects –** The study cohort comprised 10 children < 18 years of age who underwent BONEBRIDGE BCI602 implantation. The average age was of 10 (range: 5 to 14) years. All subjects had moderate conductive hearing loss with BC thresholds ≤ 25 dB HL and an average air-bone gap (ABG; 4-frequency PTA, 4PTA) > 20 dB. The patients were diagnosed with congenital microtia (6 unilateral and 4 bilateral) associated with atresia of the external auditory canal ( Table 1 ).

**Table 1:** Data analyzed in the sample of the present study

Subject number	Age at implantation (in years)	Sex	Side of the hearing loss	Tested ear	Ipsilateral AC 4PTA (in dB HL)	Ipsilateral BC 4PTA (in dB HL)
1	10.6	Female	Bilateral	Left	64	6
2	11.9	Male	Unilateral	Right	94	23
3	14.4	Male	Unilateral	Right	78	0
4	9.0	Male	Bilateral	Right	69	14
5	8.4	Female	Bilateral	Right	71	9
6	12.6	Female	Unilateral	Left	71	19
7	8.9	Male	Unilateral	Right	63	3
8	12.3	Female	Bilateral	Right	64	15
9	10.9	Male	Unilateral	Right	60	6
10	5.8	male	Unilateral	Left	70	3

**Abbreviations:** 4PTA, four-frequency pure-tone average; AC, air conduction; BC, bone conduction.

**Note:** The 4PTA was calculated from the frequencies of 0.5 kHz, 1 kHz, 2 kHz, and 4 kHz; the results presented are the means of the 4 frequencies for each subject.

**Procedure –** The present study was approved by the Ethics Committees of both clinics (Comités Institucionales de Ética en Investigación, CIEIs, no. 291/2020) and was performed according to the Declaration of Helsinki. Informed consent was obtained from the patients prior to study inclusion.

On the day of the activation of the implant system, BCI users were enrolled in the study, and tests were performed in the unaided condition. The subjects were then tested at 1, 6, and 12 months after device activation in the BCI-aided condition. After completion of this stage of the protocol, the same users were asked to stop using the BCI audio processor and instead use the aBCD for 4 weeks. At the end of this period, the measurements were repeated in the aided condition with the aBCD.

**Audiological tests –** The audiological assessment consisted of basic audiometric tests and sound field measurements in the unaided and aided conditions with the BCI and aBCD hearing systems, in an audiometric sound-attenuated room. Calibrated loudspeakers were set up at a distance of 1 m from the center of the subject's head, at ear level. For the audiological tests, the aBCD was used in program one (automatic) and with the volume at the preferred level of the patients. The SAMBA audio processor (MED-EL) was tested with the personalized fitting of the patient in the universal program. Both devices were operating with automatic beamformer, directional microphones focusing to the front in the S0 and S0N0 test setup. All devices were supplied with a new battery prior to testing. For all sound field measurements, the contralateral ear was plugged with a foam earplug and covered with an earmuff.

The auditory tests (thresholds and warble tone stimuli) were performed in a soundproof booth using a SENTIERO ADVANCED (Path Medical, Germering, Germany) audiometer in 1 center and an AC40 (Interacoustics, Middelfart, Denmark) in the other. The 4PTA was calculated from the frequencies of 0.5 kHz, 1 kHz, 2 kHz, and 4 kHz.

The word recognition score (WRS) was measured in the sound field in quiet with the speaker at 0° azimuth (S0). The percentage of words correctly recognized by the patient was assessed. Each list comprised 25 phonetically-balanced disyllabic words. 20 21

To measure speech intelligibility in noise, the speech signal (65 dB SPL) as well as the noise signal (60 dB SPL or 65 dB SPL) were provided from the front (S0N0).

**Questionnaires –** Subjective satisfaction was assessed by means of the hearing-specific Parents' Evaluation of Aural/Oral Performance of Children (PEACH) rating scale. 22 Satisfaction with the device itself was evaluated using the Audio Processor Satisfaction Questionnaire (APSQ) and a BCI/ aBCD comparison questionnaire. The PEACH questionnaire, developed by Ching and Hill, 23 comprises 13 questions about the child's behavior in everyday life in relation to a range of hearing and communication scenarios. There are 5 possible answers, ranging from never (0%) to always (75% to 100%). The APSQ questionnaire 24 consists of 21 items that refer to wearing comfort, social life, usability, and device conveniences. The responses are on a Likert scale, with 5 options ranging from never (0%) to always (100%). The custom-made BCI/ aBCD comparison questionnaire has 13 questions about the preferences of the user regarding topics like device use, wearing comfort, and sound quality. All questionnaires were thoroughly explained by the study personnel and filled out by a proxy. The proxy for a particular subject was always the same person (the child's mother, for example) throughout the study.

**Statistics –** The statistical analysis was performed using GraphPad Prism (GraphPad Software, San Diego, CA, United States) software, version 7.04. The Shapiro-Wilk test was used to test for normal distribution. The Wilcoxon signed-rank test with Bonferroni correction was applied to compare results between conditions on the following tests: 4PTA sound field thresholds, speech in quiet, speech in noise with 7 comparisons per test, resulting in a corrected *p* -value of 0.0071, and wearing time results of the APSQ questionnaire with 6 comparisons, resulting in a corrected *p* -value of 0.0083. The remaining results of the APSQ and PEACH questionnaires were analyzed by two-way repeated measures analysis of variance (ANOVA) with the Bonferroni multiple comparison test (APSQ: *F*_3,135_ = 5.37, *p* = 0.0016; PEACH: *F*_1,27_ = 55.15; *p* < 0.0001).

## Results

**Fig. 1:**
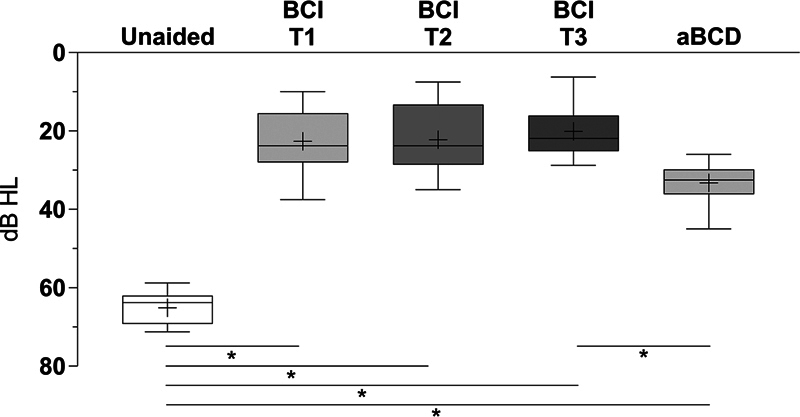
PTA4 sound field thresholds, an average of the frequencies 0.5, 1, 2, and 4 kHz, in dB HL. Bone conduction implant (BCI) at 1, 6 and 12 months after device activation (T1 – T3). Adhesive bone conduction device (aBCD). Min to max (whiskers), mean (cross) and median (line).

**Hearing thresholds –** The mean hearing threshold for the frequencies of 0.5 kHz, 1 kHz, 2 kHz, and 4 kHz (4PTA) in the unaided condition was of 65 ± 4.3 dB, which improved significantly, to 23 ± 8.1 dB, after using the BCI for 1 month ( *p* = 0.0020). Compared with the unaided results, the performance of the subjects further improved significantly after using the BCI for 6 months, with a mean 4PTA of 22 ± 8.7 dB ( *p* = 0.0020), and 12 months, with a mean 4PTA of 20 ± 7.0 dB ( *p* = 0.0020). Using the aBCD, a mean 4PTA of 33 ± 5.3 dB was measured, which was significantly higher compared with the mean 4PTA hearing threshold after using the BCI for 12 months ( *p* = 0.0020; Fig. 1 ).

**Fig. 2:**
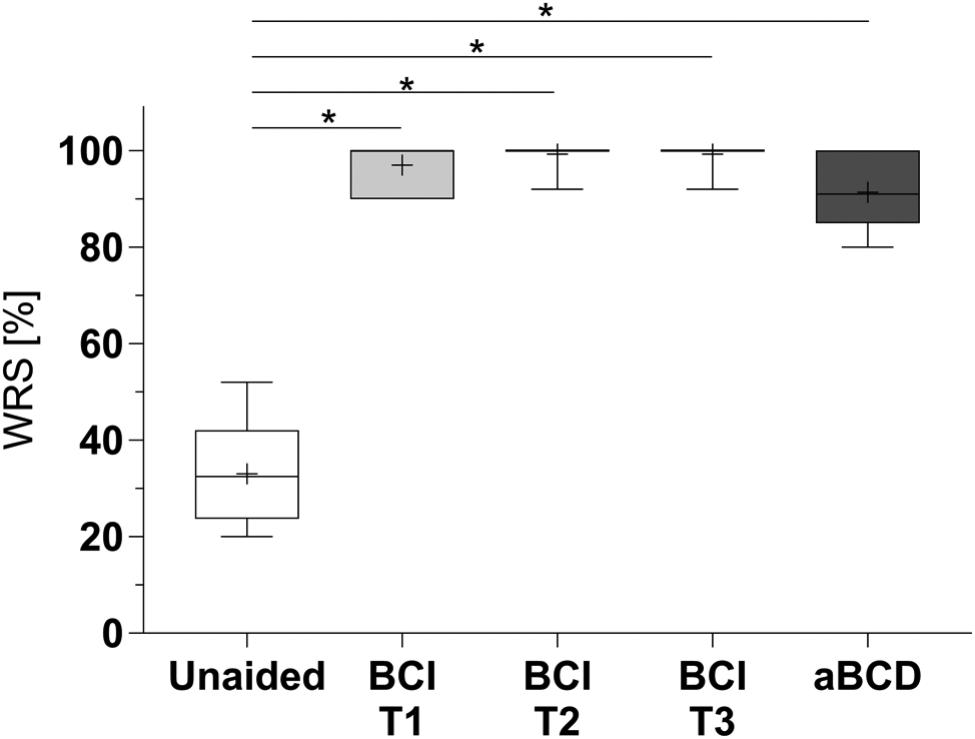
Speech in quiet. Word recognition score (WRS) in %. Bone conduction implant (BCI) 1, 6 and 12 months after device activation (T1 – T3). Adhesive bone conduction device (aBCD). Min to max (whiskers), mean (cross) and median (line).

**Speech recognition in quiet** – The speech recognition in quiet in the unaided condition presented a mean WRS of 33 ± 11%. After using the BCI or aBCD, the speech recognition improved significantly compared with the unaided condition (all; *p* = 0.0020). After using the BCI for 1, 6, and 12 months, the mean WRS values were of 97 ± 4.8%, 99 ± 2.5%, and 99 ± 2.5% respectively. With the aBCD, a mean WRS of 91 ± 7.4% could be achieved. No significant difference was found between the BCI and aBCD results ( Fig. 2 ).

**Fig. 3:**
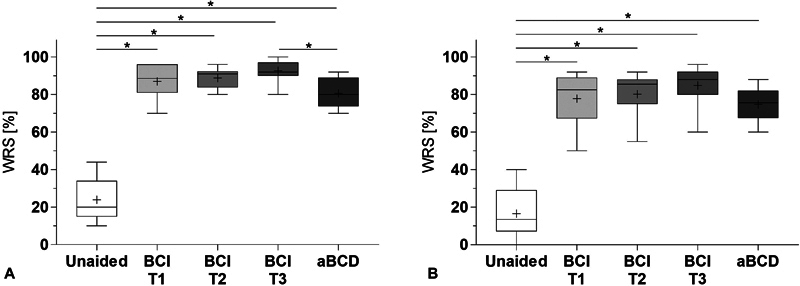
Speech in noise. Word recognition score (WRS) in % at a signal to noise ratio (SNR) of A) 5 dB SNR and B) 0 dB SNR. Bone conduction implant (BCI) at 1, 6 and 12 months after device activation (T1 – T3). Adhesive bone conduction device (aBCD). Min to max (whiskers), mean (cross) and median (line).

**Speech recognition in noise** – When speech recognition in noise was measured at a signal-to-noise ratio (SNR) of +5 dB ( Fig. 3A ), the average WRS in the unaided situation was of 24 ± 11%. Compared with the unaided condition, the speech in noise results improved significantly after using the BCI for 1, 6, and 12 months (all; *p* = 0.0020), to mean WRS values of 87 ± 9.1%, 89 ± 5.5%, and 93 ± 5.9% respectively. The mean WRS using the aBCD was of 81 ± 8.3%, which was also a significant improvement compared with the unaided condition ( *p* = 0.0020). The speech in noise result at +5 dB SNR after 12 months ( *p* = 0.0039) using the BCI was significantly better compared with the result with the aBCD. The result after 6 months using the BCI was close to statistical significance when compared with the result with the aBCD ( *p* = 0.0078).

At 0 dB SNR, the unaided speech in noise result presented a mean WRS of 17 ± 13%, which improved significantly with the BCI after 1, 6, and 12 months of use to mean scores of 78 ± 16%, 80 ± 13%, and 85 ± 10% respectively. With the aBCD, a mean WRS of 75 ± 10% was measured, which was also statistically different compared with the unaided condition (all; *p* = 0.0020). The speech in noise results using the BCI at 0 dB SNR were not statistically different from the results with the aBCD at any time point (1 month: *p* = 0.4063; 6 months: *p* = 0.2871; 12 months: *p* = 0.0195).

**Fig. 4:**
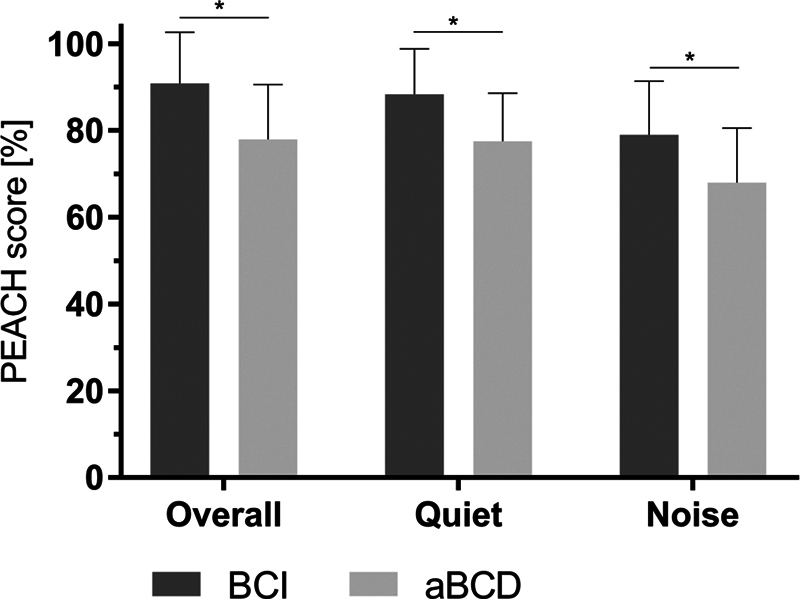
Parents' Evaluation of Aural/Oral Performance of Children (PEACH) questionnaire results, comparing the bone conduction implant (BCI) and adhesive bone conduction device (aBCD). Questions regarding communication scenarios in quiet and noise were evaluated. Standard deviation (whiskers).

**Subjective satisfaction and adherence to use –** The three questionnaires used in the present study were filled out for all ten children. The hearing-specific PEACH questionnaire was applied to evaluate the performance of the users in relation to a range of hearing and communication scenarios. Significant differences were observed between the BCI and aBCD. The mean overall score was of 91 ± 12 points for the BCI, and of 78 ± 13 points for the aBCD ( *p* = 0.0002). The mean score on the *quiet* dimension was of 88 ± 11 points for the BCI, and of 78 ± 11 points for the aBCD ( *p* = 0.0013). The mean score on the *noise* dimension was of 79 ± 12 points for the BCI, and of 68 ± 13 points for the aBCD ( *p* = 0.0011). In each case, the differences favored the use of the BCI over the aBCD. The mean daily wearing time was of 11 ± 3.0 hours per day for the BCI, and of 9 ± 2.5 hours per day for the aBCD ( Fig. 4 ).

Regarding the audio processor-specific APSQ questionnaire, no statistical difference was found in any of the domains when comparing the BCI with the aBCD. The following topics were covered by the *wearing comfort* domain: Comfort when wearing the AP, use of a phone at the processor side, physically active lifestyle with the AP, wearing glasses or head gear (cap, hat, or helmet) and the AP at the same time, and general satisfaction. In the *wearing comfort* domain, the BCI users reported a mean satisfaction rate of 79 ± 17%, and the aBCD users, 67 ± 23%. The *social life* domain consists of items regarding AP-related improvement of confidence, independence, group communication, and ease/enjoyment of social or cultural activities with the help of the device. The mean score on the *social life* domain was of 92 ± 14% for BCI users, and of 77 ± 19% for aBCD users. In the *usability* domain, the mean score of the BCI users was of 93 ± 7% and that of the aBCD users was of 80 ± 16%. The *usability* domain evaluated the following topics: AP positioning, sound location, exchanging batteries, turning the AP on and off, and maintenance. The *device conveniences* domain analyzed skin health, sweating or pressure at the AP position, and the AP falling off or malfunctioning. Using the BCI, the users reported a mean satisfaction rate of 79 ± 15%, and the aBCD users, 82 ± 16%.

**Fig. 5:**
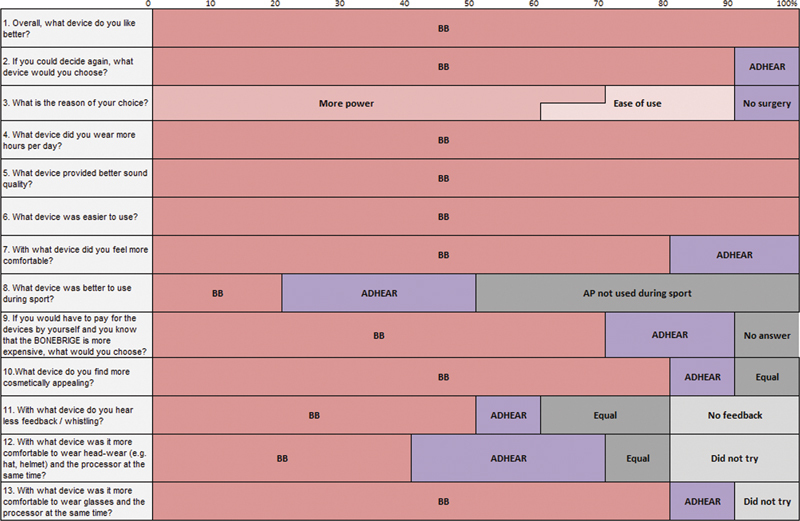
BCI/aBCD comparison questionnaire. Results of thirteen questions regarding the subject's preference, comparing the bone conduction implant BONEBRIDGE (BB) to the adhesive bone conduction device ADHEAR.

Regarding the BCI/ aBCD comparison questionnaire, favorable results for the BCI were observed in most items (Q1, 2, 3, 4, 6, 7, 9, 10, and 13). Greater dispersion in the responses was found for items Q8 (“What device was better to use during sports?”), Q11 (“With what device do you hear less feedback/whistling?”), and Q12 (“With what device was it more comfortable to wear headwear (such as hat, helmet) and the processor at the same time?”) ( Fig. 5 ).

## Discussion

The present study assessed audiological performance and subjective satisfaction in a cohort of children with moderate to severe conductive hearing loss provided with an active BCI system and compared these outcomes with those of the use if a nonsurgical aBCD in the same subjects. The study aimed to answer the question of whether or not surgical treatment is necessary by comparing treatment with a nonsurgical approach in the same patient group.

Both devices provided substantial, clinically relevant hearing rehabilitation for the patients, which is consistent with previously published outcomes. 25 26

The present study had the added value of being able to compare the hearing performance with both devices in the same group of users, reducing the possibility of bias. The audiological results between the BCI and aBCD during the first month after implant activation are comparable, although with a trend toward better performance with the BCI. After 1 year of implant use, superior results for the BCI versus the aBCD could be found in sound field thresholds (mean 4PTA: 20 dB versus 33 dB respectively; *p* = 0.0020) and in speech in noise at the SNR of +5 dB, (93 versus 81% respectively; *p* = 0.0039). A study 27 in which the audiological performance of ADHEAR was tested after 1, 6, and 12 months of use showed no improvement in the outcomes over time. Even comparing an acute test with the results after 2 months of use revealed stable audiological performance with the ADHEAR. 13 However, several studies 28 29 30 have shown that the BCI requires an acclimatization time of several months to reach its performance plateau. The reasons for the differences we have observed between both devices could lie in the different transducer design and placement, higher output with the active BCI, and transmission loss through the skin with the passive aBCD. The transducer of the aBCD is located outside and on top of the skin, transferring vibrations passively through the skin with associated signal dampening. 31 32 33 The transducer of the active implant sits in the skull bone and stimulates the bone directly. Besides the differences in output parameters of these devices, the active design of the implant enables signals to be transferred to the cochlea with minimal transfer loss. Gavilan et al. 33 (2019) compared the audiological performance of the aBCD with a passive BCI (as opposed to the active BCI used in the present study). Both systems transfer vibrations passively through the skin, and comparable results between the aBCD and the passive transcutaneous BCI were reported.

Good, aided speech perception is particularly important for children, especially in noisy environments such as school. Research 34 35 36 has shown that even untreated unilateral hearing loss negatively affects language development, communication skills, academic progress, and social/emotional development. Although more costly and invasive, an active BCI appears to be the best device for hearing rehabilitation in these patients.

Percutaneous bone-anchored hearing aids (BAHAs) also directly stimulate the bone. However, device-associated skin complications and related inconveniences have been regularly reported. 37 38

We have seen a ceiling effect in WRS results in quiet with the BCI, as well as in some aBCD cases. Therefore, additional test setups like speech in noise were needed to evaluate performance differences. A speech in quiet test at a lower presentation level would have been a valuable addition to the standard 65 dB SPL. The relatively small sample size and the restrictive inclusion criteria are further limitations of the present study. The indication of the BCI is for up to 45 dB HL BC hearing loss, and both devices could be used for patients with unilateral deafness if hearing of the contralateral ear would fall within the indication criteria. However, the population of the present study was chosen to facilitate optimal comparability of both systems within the same subjects.

The questionnaire results showed high subjective satisfaction with the aBCD, which is consistent with the published results of a comparable patient group. 27 Although most results with the APSQ audio processor-specific questionnaire were slightly better for the BCI compared with the aBCD, there was no statistically significant difference. However, the BCI/aBCD comparison questionnaire provided a clearer picture, as users had to choose between the BCI or aBCD or report equal performance. On the individual level, the BCI was mostly chosen as the preferred solution due to its superior output. One user preferred the aBCD as no surgery is required. In addition, the subjects preferred the BCI to the aBCD in terms of sound quality, cosmetic appeal, and ease of use. The BCI was also mostly chosen as the better option for those who wear glasses and the AP at the same time. However, several patients preferred the aBCD during sports. It is possible that users perceive the BCI processor to be more fragile and costly to repair than the aBCD.

Regarding hearing-specific subjective satisfaction, the results of the PEACH questionnaire revealed a clear superiority of the BCI over the aBCD, and they are in line with the audiological results comparing both systems. As another measure of overall satisfaction, the wearing time results support the subjective satisfaction findings, as the aBCD was used sufficiently (9 hours per day); however, the BCI audio processor was used 2 hours longer on average. Although the active BCI was superior to the passive aBCD in most objective and subjective results, the overall good results and high adherence to use reinforce how useful the aBCD can be for this group of patients. Lastly, there were no adverse events reported with either of the devices: both were well tolerated by the patients, and no problems were reported during the course of the present study.

## Conclusion

In the sample of the present study, hearing performance with the passive transcutaneous device was clinically sufficient and, regarding certain results, comparable to the active BCI. However, superiority of the implant was shown in terms of quality of life and after device acclimatization in speech in noise. Thus, the aBCD should be considered an alternative in cases in which surgery is not desired or not possible.
